# Formation and purification of tailored liposomes for drug delivery using a module-based micro continuous-flow system

**DOI:** 10.1038/s41598-017-11533-1

**Published:** 2017-09-21

**Authors:** Nikolay Dimov, Elisabeth Kastner, Maryam Hussain, Yvonne Perrie, Nicolas Szita

**Affiliations:** 1 Department of Biochemical Engineering, University College London, London, WC1H 0AH UK; 2 Aston Pharmacy School, School of Life and Health Sciences, Aston University, Birmingham, B4 7ET UK; 3 Strathclyde Institute of Pharmacy and Biomedical Sciences, University of Strathclyde, Glasgow, G4 0RE Scotland

## Abstract

Liposomes are lipid based bilayer vesicles that can encapsulate, deliver and release low-soluble drugs and small molecules to a specific target site in the body. They are currently exploited in several nanomedicine formulations. However, their development and application is still limited by expensive and time-consuming process development and production methods. Therefore, to exploit these systems more effectively and support the rapid translation of new liposomal nanomedicines from bench to bedside, new cost-effective and scalable production methods are needed. We present a continuous process flow system for the preparation, modification and purification of liposomes which offers lab-on-chip scale production. The system was evaluated for a range of small vesicles (below 300 nm) varying in lipid composition, size and charge; it offers effective and rapid nanomedicine purification with high lipid recovery (> 98%) combined with effective removal of non-entrapped drug (propofol >95% reduction of non-entrapped drug present) or protein (ovalbumin >90% reduction of OVA present) and organic solvent (ethanol >95% reduction) in less than 4 minutes. The key advantages of using this bench-top, rapid, process development tool are the flexible operating conditions, interchangeable membranes and scalable high-throughput yields, thereby offering simultaneous manufacturing and purification of nanoparticles with tailored surface attributes.

## Introduction

Liposomes are a well-established formulation strategy to improve drug delivery and enhance therapeutic outcomes for a range of drugs, such as pharmaceuticals, biopharmaceuticals, and vaccines. Due to their bilayer vesicle structure, which is akin to natural cells, liposomes are able to incorporate drugs both within their aqueous core and their lipidic bilayers. Through such means, the pharmacokinetics of a drug can be controlled and dictated by the liposomal delivery system rather than the drug attributes. This has allowed the development of a range of clinically approved liposome-based medicines including DOXIL/Caelyx® (doxorubicin), AmBisome® (amphotericin B) and Daunoxome® (daunorubicin), which when combined have an annual market revenue of approximately $100 million. However, despite these advantages, their wider application is limited by their complex and costly production requirements. Currently, manufacturing methods include the use of solvent injection, reverse-phase evaporation and emulsification methods^1^. Such methods have the disadvantage of involving multi-step processes, often adopt large amounts of organic solvents and are limited to batch-release processes. Furthermore, a crucial attribute to an effective liposomal drug system is the vesicle size range, which can be controlled the production method, e.g. sonication (20–40 nm)^2^, extrusion (70–415 nm)^3^ or high-pressure homogenisation (20–140 nm)^4^; and, more recently, microfluidic mixing (20–80 nm)^5,6^ or flow focusing (50–150 nm)^7^. Upon administration, the pharmacokinetic profile and fate of liposomes is dictated by their size and therefore controlling particle size and polydispersity (PDI) is a key issue in their manufacturing and a key parameter in the product specifications. To produce liposomes in a controlled size range, downsizing through extrusion or homogenisation is often adopted. This adds further steps to the manufacturing process and exposes the liposomes and drug constituents to harsh and potentially detrimental processing conditions. To address these issues, and allow the wider adoption of liposomal systems to improve health-care, new methods in liposome manufacture are therefore required.

Microfluidic devices operate with small volumes, offer exquisite control over the fluid flow^8,9^, and make efficient use of materials, reagents and energy^10^. These advantages have been applied for the reproducible formation of liposomes with uniform size distribution^1,6,11^. Typically, liposome formation occurs at the interface of an aqueous and a solvent phase, containing lipid molecules^12^, and microfluidic devices are well suited to establish and finely control such interfaces. In hydrodynamic flow focusing (HFF) devices, for example, where the solvent phase is microinjected in between two co-flows of aqueous buffer, liposomes with well-controlled size distributions can be assembled ^6,13^. Furthermore, by changing the ratio between the flow rates of the aqueous buffer and organic phase, the concentration of lipid molecules in the organic phase, or by adapting the channel geometry, the size of the liposome can be finely prescribed^14,15^. Scaling up of HFF devices, however, is difficult which constrains the amount of liposomes that can be produced per unit time^16^. In contrast, devices based on chaotic advection micromixing^11,17^ are more suitable for high throughput production of liposomes. Lipid nanoparticles with sizes between 20 and 50 nm were reported using a staggered herringbone mixer (SMH) by varying triglyceride ratios^5^. More recently, Kastner *et al*. used the same SMH to prepare liposomes encapsulating propofol^18^, a poorly water-soluble drug. These works demonstrate the potential application of microfluidics for the rapid, reproducible and size-controlled formation of drug-loaded liposomes.

Purification remains a significant hurdle in the development of liposomal products. Irrespective of which production method is adopted, non-entrapped contaminant molecules, small molecule drugs or proteins must be removed from the final liposome product. Separation is typically achieved by filtration^19,20^ or ultra-centrifugation, which can be challenging for the large-scale purification. Other possible routes for removal of non-encapsulated material include dialysis, gel-permeation chromatography, ion-exchange chromatography, and size exclusion chromatography. However, these processes are time-intensive and can furthermore diminish product yield by column equilibration, which dilutes the final liposomal product, even with size exclusion chromatography^21^.

To address these issues of post assembly refinement, here we investigate a ‘lab-on-chip’ module-based microfluidic manufacturing and purification system for the production of liposomes. In contrast to previously reported on-chip devices by other groups and by us^5,18,22,23^, where lengthy dialysis procedures for removal of non-entrapped drug and solvent residues were required, we present a novel continuous microfluidic liposome production and purification process train which generates purified liposomal products in less than 4 minutes. Furthermore, the purification step is based on a tangential flow-filtration device with an easily exchangeable membrane, allowing therefore the purification of a broad variety of liposome formulations. This robust process train facilitates the identification of prospective formulations, optimal operating conditions and scale-up parameters, whilst significantly reducing the time required for developing versatile adjuvant and drug delivering systems.

## Results

In order to obtain a continuous microfluidic liposome production and purification process train, we first characterised a tangential flow filtration (TFF) device^24^ and its purification capabilities for a variety of liposome formulations using lipids as outlined in Table 1.

**Table 1:** Lipids investigated in this study.

Lipid	Application	Reference
Dimethyldioctadecyl ammonium bromide(DDA)	Vaccine adjuvant,cationic head group, uptake of vaccine antigens to antigen presenting cells	Smith Korsholm *et al*.^34^ Christensen *et al*.^35^
Trehalose 6,6-dibehenate(TDB)	Synthetic immunstimmulator derived from the membrane of mycobacterium	
1,2-dioleoyl-sn-glycero-3-phsphoethanolamine(DOPE)	Fusogenic helper lipid, available in the commercial Lipofectin™ transfection reagent	Henriksen-Lacey *et al*.^36^
1,2-dioleoyl-3-trimethylammonium-propane (DOTAP)	Cationic lipid often used in transfection	
Egg Phosphatidylcholine(PC)	Neutral head group, drug delivery	Senior and Gregoriadis^37^ Gregoriadis and Senior^38^
1,2-Dipalmitoyl-*sn*-glycero-3-phospho-*rac*-(1-glycerol)(DPPG)	Negative charged head group, drug delivery	Oku *et al*.^39^ Kirby *et al*.^40^
1,2- Dipalmitoyl-*sn*-glycero-3-phosphocholine(DPPC)	Neutral head group, drug delivery	
Cholesterol (Chol)	Added for membrane stabilization, known to effect drug encapsulation efficiency in bilayer and aqueous core	Senior and Gregoriadis^37^ Kirby *et al*.^40^

### On-chip purification of liposomes for determination of the range of operational transmembrane pressures

**Figure 1:**
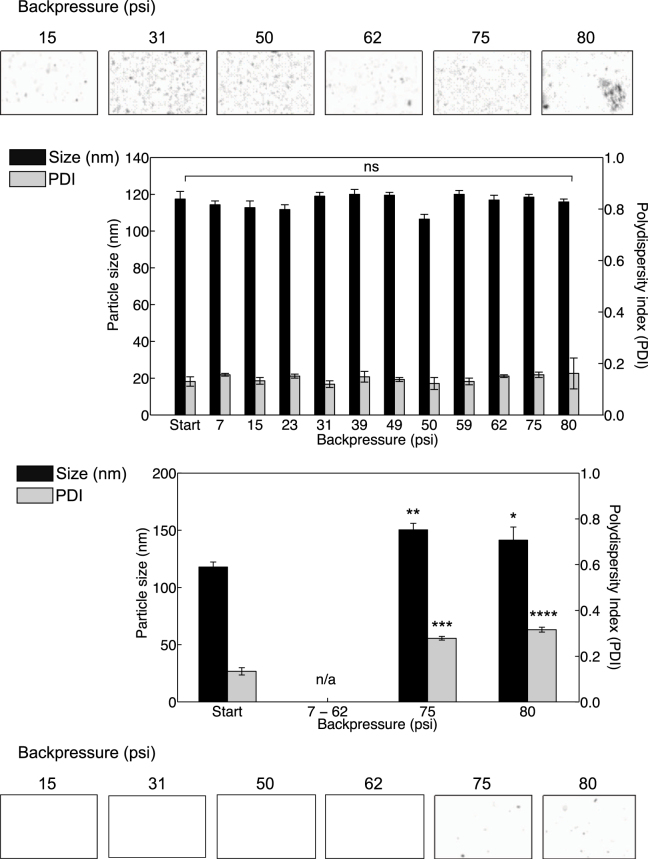
Particle size and polydispersity as a function of increasing backpressures in the TFF system as collected on the retentate side of the membrane. Images from NTA analysis, verifying particles in permeate (top) and retentate (bottom) stream at increasing backpressures. Particles were found in the permeate at backpressures exceeding 75 psi. All experimental datasets are presented as mean and standard deviation (mean ± s.d.) resulting from three independent runs (n = 3).

To identify the operational backpressure which yields the maximal recovery, we introduced in to the TFF device a commonly used formulation of neutral liposomes (PC:Chol; 1:1 molar ratio; Table 1). By varying the flow rates, and by implementing capillaries with different inner diameters and lengths downstream of the TFF device, we investigated backpressures from 7 to 80 psi (Table 2). To assess the liposome retention on the retentate side of the membrane (the volume of the liquid, which does not pass through the membrane), we collected samples from both the retentate and permeate (the volume which passes through the membrane), and measured the liposomal size and polydispersity index (PDI) at each of the backpressure conditions. The results (Fig. 1) show that there was no significant change in the size (approximately 115 nm) and PDI (0.15) of the liposome suspension in the retentate across the pressure range tested. However, at backpressures of 75 psi and 80 psi, particles were detected in the permeate as confirmed by qualitative image-based nanoparticle tracking analysis NTA (Fig. 1). To confirm membrane integrity and exclude membrane damage as a possible cause for the liposome transferred into permeate, a leak-test applying pressures higher than 80 psi was run and confirmed the membrane was intact. This suggests that at pressures above 75 psi, the TFF device was unable to retain liposomes and these were being pushed across the membrane into the retentate.

**Table 2:** Backpressures and flow rates through the Tangential Flow Filter (TFF) that were investigated in this study.

Backpressure (psi)	7	15	23	31	39	49	50	59	62	75	80
Flow rate (mL min^−1^)	0.01	0.02	0.03	0.1	0.05	1	0.3	2	2.5	0.5	0.1
Capillary I.D. (μm)	50	50	50	63	50	100	63	100	100	63	50
Capillary length (mm)	50	50	50	50	50	50	25	30	25	25	50

Liposomes in solution were fed into the TFF device at flow rates ranging between 0.01 and 2.5 mL min^−1^. Backpressure was attained by connecting a restrictive capillary with selected (I.D.) and/or length on the retentate side of the TFF outlet.

### The effect of filtration on particle characteristics of cationic and anionic liposomes

**Figure 2:**
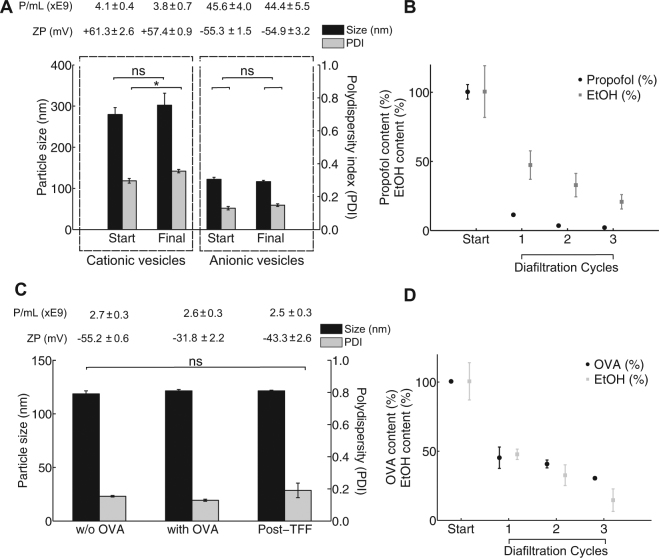
(**A**) Vesicle size, polydispersity (PDI), zeta potential (ZP) and particle concentration (P/mL) for cationic (DDA:TDB) and anionic (DPPG:DPPC:Chol) liposomes before and after the TFF purification. (**B**) Images from NTA show vesicles present in the retentate side only. (**C**) Propofol and ethanol removal achieved over three diafiltration cycles for anionic liposomes (DPPG:DPPC:Chol), expressed as a percentage of the initial amount of contaminants present.

After proving that neutral PC:Chol liposomes were retained, the application of the TFF to purify cationic (DDA:TDB; 8:1 molar ratio; Table 1) and anionic liposomes (DPPC:Chol:DPPG; 4:4:1 molar ratio; Table 1) was assessed (Fig. 2). The TFF device was first challenged with a batch-formulated cationic liposomal adjuvant (DDA:TDB) in three diafiltration cycles with buffer replenishment after each cycle, compensating for the volume of liquid passing into permeate. At a flow rate of 1 mL min^−1^, the backpressure was 49 psi (capillary length 50 mm, and I.D. 100 µm, Table 2), yielding a calculated flow rate of water through the cellulose membrane, Q_transmemb_, of 0.25 mL∙min^−1^ (based on a linear extrapolation from supplier data; 16 mL min^−1^ cm^−2^ for 14.5 psi).

With the cationic liposomes, the particle concentration of liposomes was 4.1 × 10^9^ P/mL, which reduced to 3.8 × 10^9^ P/mL at the end of the third cycle (Fig. 2A) confirming the yield from the filtration process was 93% for the cationic liposomes. With these systems the liposomal size (approximately 300 nm) and cationic nature (approximately 60 mV) were not notably influenced by the filtration process (Fig. 2A). Furthermore, NTA analysis showed that there were no cationic liposomes detected in the permeate.

Similar results were demonstrated with anionic liposomes; filtration of batch-formulated anionic liposomes (DPPC:Chol:DPPG) using three diafiltration cycles produced no notable changes in terms of vesicle size (approximately 120 nm), PDI (0.14 to 0.15), ZP (−55 mV) and particle concentration (4.4 to 4.6 × 10^10^ P/mL) (Fig. 2). As with the neutral and cationic liposomes, NTA analysis in each diafiltration cycle verified that no liposomes were present in the permeate.

### Purification of non-incorporated drugs from liposome formulations

Having successfully demonstrated the capability of the TFF to retain a wide range of different liposome systems, we then focused on investigating the efficiency of the TFF to purify the liposomal nanomedicines and remove non-incorporated drug (RC membrane, 10 kDa cutoff). To study drug removal, propofol (1 mg mL^−1^) was added to a suspension of negatively charged liposomes (DPPC:Chol:DPPG) in aqueous solution containing 20% (v/v) ethanol residual solvent levels found after liposome production by microfluidics prior to purification. Propofol, was employed as it has previously been studied as a low-solubility drug solubilised within liposomes. The TFF was shown to effectively purify the liposomes by removing both the solvent and non-incorporated drug with 90% of the non-incorporated propofol being removed in the first diafiltration cycle, with a further removal of 80% in the second diafiltration run, and a further 60% after the third diafiltration cycle (Fig. 2B). Thus, after three cycles only 1% of the ‘free’ non-incorporated drug remained within the formulation (Fig. 2B). Simultaneously, the TFF system removed the ethanol which had been used for the liposome formulation (Fig. 2B). Ethanol was reduced by approximately 50% in the first diafiltration cycle, and after three diafiltration cycles the residual ethanol concentration was 3% (v/v) (Fig. 2B).

### Purification of ‘free’ protein from liposome formulations

To investigate the removal of non-entrapped protein from liposome formulations, both cationic (DDA:TDB) and anionic liposomes (DPPC:Chol:DPPG) were considered given that electrostatic interactions between cationic liposomes and anionic proteins is exploited in the loading of antigens to liposomal adjuvant systems. Therefore both liposome systems were mixed with ovalbumin (OVA; 100 μg mL^−1^). At a flow rate for the retentate of 2.5 mL min^−1^, the backpressure was 62 psi (capillary length 25 mm, and I.D. 100 µm, Table 2), yielding a calculated flow rate of water through the PES membrane (MWCO 300 kDa), Q_transmemb_, of 1.69 mL·min^−1^ (based on a linear extrapolation from supplier data; 58 mL min^−1^ cm^−2^ for 10 psi). Thus, from 2.5 mL initial sample only 0.81 mL remain in the retentate fraction, and 1.69 mL pass through the membrane. The theoretical volume of permeate accounted for 67% of the initial liquid.

**Figure 3:**
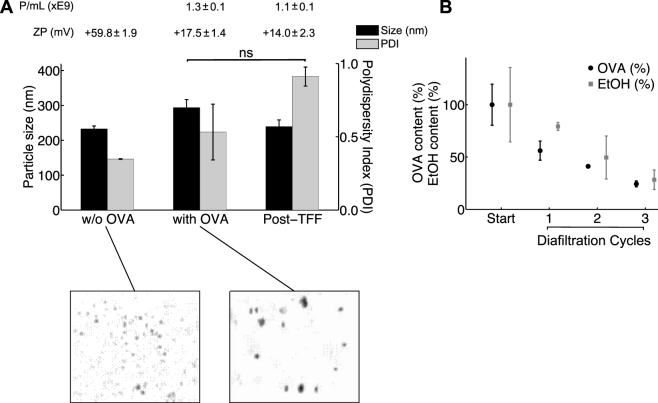
Vesicle size, polydispersity (PDI), zeta potential (ZP) and particle concentration (P/mL) for (**A**) anionic liposomes (DPPG:DPPC:Chol) and (**B**) cationic liposomes (DDA:TDB) prior and post OVA-addition (ovalbumin, 100 μg mL^−1^), and particle characteristics after the TFF purification. Protein (ovalbumin) and ethanol removal achieved over three diafiltration cycles for (**C**) anionic and (**D**) cationic liposomes, expressed as a percentage of the initial amount of contaminants present. All experimental datasets are presented as mean and standard deviation (mean ± s.d.) average of three independent runs (n = 3).

Given the anionic nature of OVA at the pH range used, electrostatic interactions with the cationic but not the anionic liposomes occurred. Indeed, anionic liposomes maintained a similar size (approximately 120 nm) after the addition of OVA (Fig. 2C); however, for the cationic liposomes, the electrostatic interactions with the anionic OVA resulted in aggregation and in an increased vesicle size from around 220 nm to 300 nm, and a drop in their cationic nature from 59.8 ± 1.9 mV to 17.5 ± 1.4 mV (Fig. 3A).

After the addition of protein, both systems were subjected to three diafiltration cycles. The size and PDI of both the anionic (Fig. 2C) and cationic (Fig. 3A) liposomes were not significantly altered through the course of the diafiltration cycles and particle recovery was 87% and 96%, respectively. Protein (OVA) and residual ethanol was removed into the permeate stream over the three diafiltration cycles, with a final removal of 70% of the free protein and 95% of the solvent with the anionic liposomes (Fig. 2D). Similar results were achieved with the cationic liposome systems (Fig. 3B); by using TFF purification, ethanol residues were reduced to 4% (v/v) of the starting value and 75% protein was removed (Fig. 3B) after three diafiltration cycles. The reduced levels of protein removed from the cationic system were a result of protein loading onto the surface of the liposomes due to electrostatic interactions (but not related to the filtration process in the TFF).

### Micro continuous-flow system for production, modification and purification of liposomes

**Figure 4:**
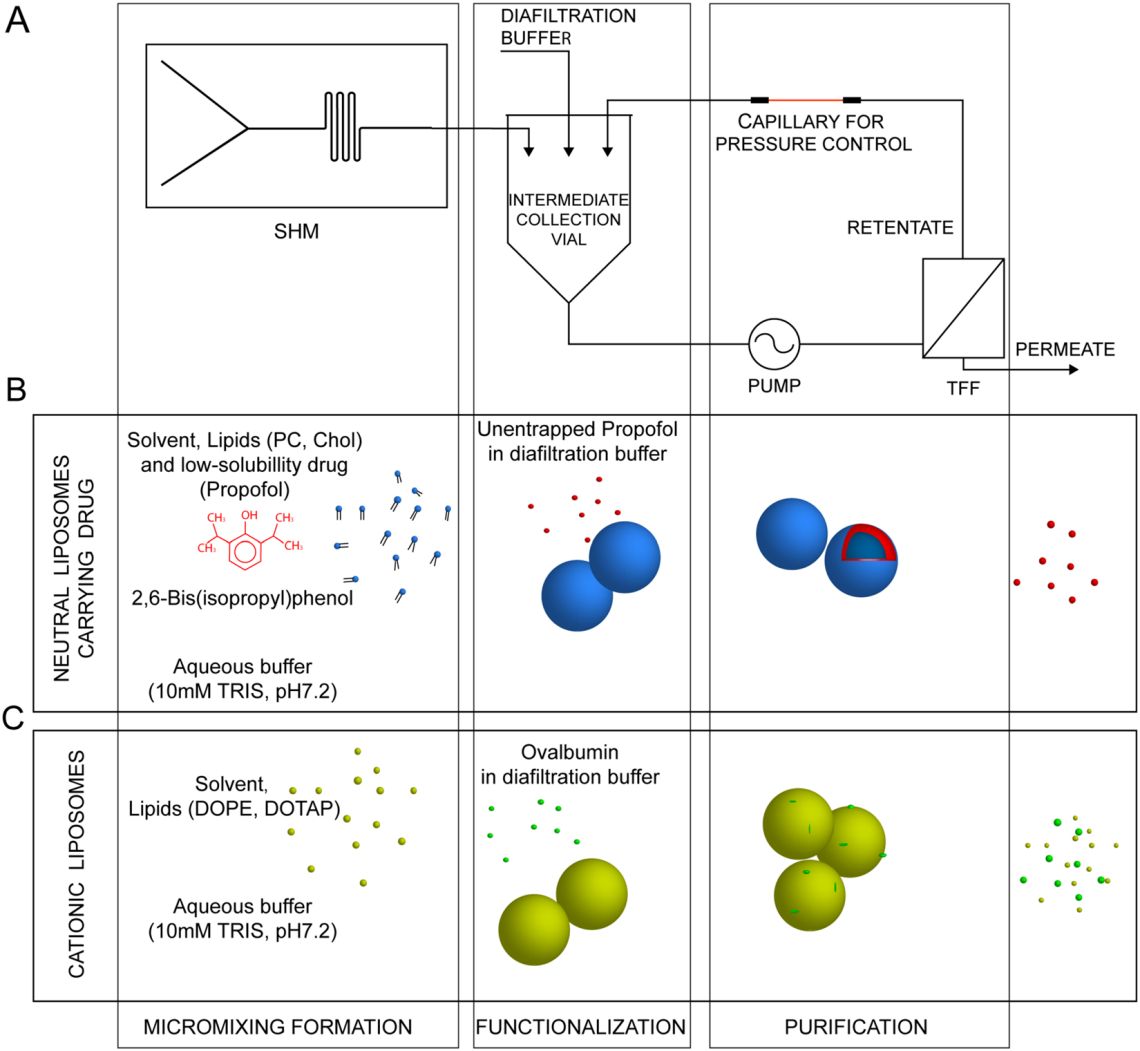
(**A**) Schematic overview of the module-based microfluidic system. Liposomes were manufactured with a **S**taggered **H**erringbone **M**ixer (SHM) upstream and flowed through the **T**angential **F**low **F**iltration (TFF) device for consecutive purification. (**B**) Schematic overview of the formation of liposomes loaded with a low-solubility model drug, *i*.*e*. propofol. Vesicle assembly and drug loading are performed with a SHM, and non-entrapped (free) drug is removed from the mixture by consecutive filtration inside the TFF system. (**C**) Schematic overview of the formation of liposomes loaded with a model protein, ovalbumin (OVA). Vesicle assembly is performed with a SHM, with post-assembly protein addition; non-entrapped (free) protein is removed by consecutive diafiltration cycles inside the TFF system.

**Figure 5:**
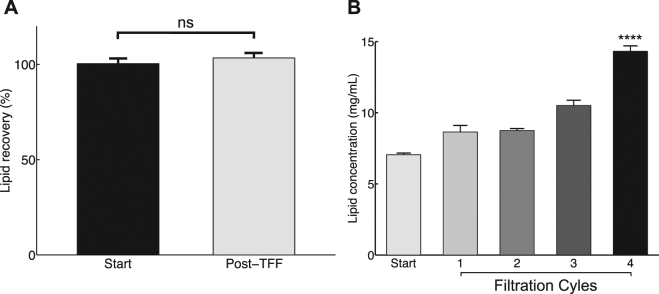
Lipid recovery in the continuous liposome factory-on-a-bench for (**A**) lipid recovery after four diafiltration cycles. (**B**) Lipid concentration in four concentration cycles, related to the initial amount of lipids present prior to the concentration cycles. All experimental datasets are presented as mean and standard deviation (mean ± s.d.) average of three independent runs (n = 3).

Having established the efficacy of the TFF purification system, the next stage was to develop a continuous manufacturing and purification process for liposomes. To achieve this, a staggered herringbone micromixer (SHM) was employed to generate the liposomal systems and directly feed the TFF device with the liposomes. To optimise the throughput for each of the two devices separately, and to independently control the flow rates, an intermediate collection vial was used (Fig. 4). Furthermore, the intermediate collection vial allowed purification of the liposomes in diafiltration mode. After each diafiltration cycle, fresh buffer was added manually into the intermediate vial to compensate for the volume passing through the membrane into the permeate. Continuous production (Fig. 4) was demonstrated by using 1) neutral liposomes (PC:Chol, 4:1 molar ratio) with propofol and 2) cationic liposomes (DOPE:DOTAP, 1:1 molar ratio; Table 1) loaded with surface-complexed protein. Lipid recovery after 4 diafiltration cycles remained at 100%, matching the initial amount of lipids present prior to TFF (Fig. 5A). Without buffer replenishment, the system performed concentration cycles for formulations (Fig. 5B). Within four cycles, the concentration of lipids measured in the retentate doubled (Fig. 5B). This was due to a 50% reduction in volume as the overall quantity of lipids in the retentate remained constant, matching the lipid content after the SHM (but before the TFF).

### Continuous manufacture and purification of liposomes with bilayer loaded drug

This system was then applied for the continuous manufacture and purification of liposomes incorporating propofol (Fig. 4B). Liposome (PC:Chol) production and drug encapsulation were performed in a staggered herringbone mixer (SHM), operated with a volumetric flow rate of 2 mL min^−1^ and a 1:3 solvent:aqueous buffer ratio. The resulting liposomes were 50 nm in size with a PDI of 0.3 (Table 3) in line with previously reported studies^25^. Using the continuous manufacturing set up (with three diafilitrations), liposomes were therefore both manufactured and purified. This continuous system was able to produce a purified liposome product incorporating 51 mol% propofol (in line with previously reported drug loading achieved using a 2 step manufacture and purification process based on dialysis^18^), with clinically acceptable ethanol levels (3% (v/v); Table 3). Furthermore, liposomes manufactured and purified in this continuous systems retained their physico-chemical attributes and were not significantly different in size, nor PDI from those not subjected to TFF purification (Table 3). Examples of electron microscopy images of liposomes are shown in Supplementary Figures S1 and S2.

**Table 3:** Continuous purification of PC:Chol liposomes loaded with propofol.

	Liposome with drug after SHM	Liposome with drug after three passes through the TFF^*^
Size (nm)	51.4 ± 2.1	61.2 ± 13.2
Polydispersity	0.29 ± 0.013	0.33 ± 0.09
Loading (mol%)	N/A	51.0 ± 4.0
Effec. ethanol (% v/v)	16.1 ± 3.9	3.1 ± 1.5

Propofol and lipids were included in the ethanol stream. Liposome formation and drug encapsulation was performed in a staggered herringbone mixer (SHM), operated with a total flow rate of 2 mL min^−1^ and a ratio of 1:3 ethanol:aqueous solution. The results are presented as mean and standard deviation (mean ± s.d.) resulting from three independent runs (n = 3), N/A = not applicable.

^*^After each pass a volume of pure buffer was added to compensate for permate and maintain constant volume of retentate.

To compare the characteristics and drug loading of PC:Chol liposomes loaded with propofol, the same formulation was prepared using hand-held extrusion (10 passes through a 400 nm, 200 nm, 100 nm and final 50 nm pore size filters). Whilst this method of liposome manufacture was not the main focus of this study and could be further optimized, again these liposomes were effectively purified to remove free drug using TFF (with drug loading of 3.6 ± 0.38 mol %; data not shown) and the liposome size and PDI remained unchanged by TFF purification (107.9 ± 14.1 nm and 109.9 ± 19.0 nm with PDI values of 0.17 ± 0.10 and 0.34 ± 0.06 pre and post TFF purification respectively, results not shown).

### Continuous manufacture and purification of cationic liposomes with adsorbed protein

The lab-on-chip micro continuous-flow system was next challenged with the production of cationic (DOPE:DOTAP) liposomes, which were modified with added ovalbumin in the intermediate connection vial and finally subjected to purification (Fig. 4C). Lipids were included in the ethanol stream and liposome formation was performed using the SHM, which operated at 2 mL min^−1^ and a 1:3 solvent:aqueous buffer ratio. The outflowing liposome solvent mixture was collected in the intermediate collection vial after 1 minute of SHM operation, and analysed (size, PDI, ZP). The resulting liposomes had a size of 62.8 ± 1.9 nm, PDI of 0.4 ± 0.02 and ZP of 84 ± 3.5 mV prior to addition of OVA. Then ovalbumin was added to the intermediate vial, resulting in vesicles with a larger size (88.5 ± 5.7 nm), unaltered polydispersity (0.45 ± 0.01), and reduced ZP (43.6 ± 1.6 mV) (Table 4), again as a result of interactions between the cationic liposomes and the anionic protein. After manufacture and purification on the system, liposomes were unaltered in size (89.3 ± 10.9 nm; PDI 0.42 ± 0.02) and had an increased ZP (69.2 ± 6.1 mV) (Table 4), presumably through the purification and removal of 74% ‘free’ protein from the system. Residual solvent levels were also reduced to clinically acceptable levels (4%; Table 4). Examples of electron microscopy images of liposomes are shown in Supplementary Figure S1.

**Table 4:** Continuous purification of DOPE:DOTAP liposomes loaded with protein (ovalbumin).

	Liposome w/o OVA after SHM	Liposome with OVA in collection vial	Liposome with OVA after three passes through TFF^*^
Size (nm)	62.8 ± 1.9	88.5 ± 5.7	89.3 ± 10.9
Polydispersity	0.44 ± 0.02	0.45 ± 0.01	0.42 ± 0.02
Zeta potential (mV)	83.9 ± 3.5	43.6 ± 1.6	69.2 ± 6.1
Loading (%)	N/A	N/A	23.9 ± 0.8
Effec. ethanol (% v/v)	N/A	15.0 ± 6.9	4.1 ± 1.5

The lipids were included in the ethanol stream and liposome formation was performed in a SHM, operated at 2 mL min^−1^ and a ratio of 1:3 solvent:aqueous solution. Protein was added post-liposome formation. OVA = ovalbumin, N/A = not applicable.

^*^After each pass a volume of pure buffer was added to the retentate, keeping the level of liquid constant.

## Discussion

We successfully investigated the region of operation for the Tangential Flow Filtration (TFF) device with various liposome formulations and confirmed the upper limit of operational pressure for the presented purification system to be 75 psi. A pressure range between 5 and 80 psi is a common backpressure implemented in industrial filtrations^26^ which is virtually identical to our TFF. During pressure tests, the membrane remained intact throughout, and therefore it can be considered that the measured backpressure equaled the transmembrane pressure inside the TFF. This transmembrane pressure could be adjusted accordingly using the data available in Table 2; alternatively, it could be calculated from Hagen Poiseuille’s equation. Based on these findings we determined the optimal operational transmembrane pressure of 62 psi, which corresponded to a maximum flow rate of 2.5 mL min^−1^ through a restrictive capillary with an internal diameter of 100 µm and a length of 25 mm. At this flow rate, our sample (2 mL) took less than a minute (~48 s) to run through the system, which shows a distinct advantage over the current methods that require lengthy bench-top, post-synthetic dialysis^22^. At high shear rates, drug release from liposomes can be a problem. However, the calculated average shear rate at the maximum flow rate of 2.5 mL min^−1^ inside the retentate channel is approximately 590 s^−1^ (Supplementary Information S3). This value is lower than previously reported shear rates^27^ of 800 s^−1^ for which no influence on the permeability or integrity of the liposome membranes was found. Furthermore, the flow rates matched those previously applied for liposome manufacturing using a device with a SHM^5,25^. Thus, we proved that a SHM can be coupled directly with the TFF, and that we could generate and purify liposomes in a continuous mode without any losses into the permeate. Overall our results show that our filtration system can be implemented for multistage purification of a broad range of liposomal products.

Backpressures of 75 psi and higher, however, led to losses of liposomes through the intact cellulose membrane into the permeate. One possible explanation could be that of particle extrusion across the membrane at these high pressures. It is well known that liposomes can undergo extrusion through cylindrical pores in membranes. Industrial scale extrusion tends to use higher lipid concentration than in our current study and adopts higher pressures ranging between 100–700 psi. However, extrusion of liposomes is system dependent; polycarbonate filters are used at pressures less than 100 psi, and low lipid concentrations require lower pressure^28^. Therefore, to avoid extrusion, a backpressure of 75 psi was adopted as the critical cut off value. Membrane characteristics also play an important role for liposome recovery as they influence the flux from the retentate to the permeate. The calculated transmembrane flow was 0.32 mL min^−1^ (or 12.8% of the total flow rate, TFR) for a hydrophilic membrane with a pore size of 0.22 µm, at a backpressure of 62 psi and nominal flow rate of 2.50 mL min^−1^ (retentate). In contrast, for the same backpressure and same nominal flow rate, a membrane with a 0.45 µm pore size resulted in a transmembrane flux of 1.69 mL min^−1^, corresponding to 67.6% of the retentate inflow. Furthermore, the presented setup demonstrates that a range of capillaries with varying inner diameter and length can be applied to control the backpressure and the dilution or concentration rates of the system, allowing to tailor resulting flow rates and to adjust throughputs.

Having established optimal operational conditions of our TFF device, its purification capacity for the removal of non-incorporated hydrophobic drug (propofol) and residual ethanol was studied. Over three diafiltration cycles, the quantity and quality of liposomes were preserved after purification for the anionic vesicles. For the cationic liposomes, there was a small increase in polydispersity, but no significant increase in liposome size (Fig. 2A). The propofol content decreases much faster in comparison to the ethanol (Fig. 2B), with the hydrophilic membrane (0.22 µm pore size). This could potentially be due to capillary action that channels the separation of lipophilic propofol^29^ (Log K_ow_ = 3.79) through the membrane. The ethanol content was the critical factor, which determined the required number of diafiltration cycles. After three diafiltration cycles, only 1% of non-incorporated propofol and 3% residual ethanol remained within the liposomal suspension with no changes in liposome physico-chemical attributes or concentration (Fig. 2) demonstrating the ability of this system to provide liposomes purified to a level as would be expected for a therapeutic product.

In terms of removal of non-associated protein from liposomes, as might be required for liposomal adjuvants or biological therapeutics, protein removal is challenging because high concentrations of protein can lead to membrane fouling due to protein-membrane interactions^24^. Such protein-membrane interactions occur due to electrostatic repulsion forces. Similar to propofol removal, the dilution by replenishing with fresh buffer, and subsequent filtration facilitates the reduction in concentration of free protein in the retentate. Purification therefore occurs as a result of two cumulative effects: one from the separation at the membrane and the other from the dilution of the retentate. As demonstrated, separation can be controlled by adjusting flow rates and restrictive capillary sizes; also by varying the amount of liquid that is replenished after each diafiltration cycle. In our results, the volume amounted to the volume of the permeate, thus maintaining constant the amount of liquid circulating in lab-on-chip purification system.

The tolerated levels of free protein depend on the requirements implied by the target application of the liposomes and the number of diafiltration cycles can be adjusted accordingly to match those criteria for purity. A particular focus in the delivery of protein antigens is the use of cationic liposomes, with electrostatic attractive forces dominating and often leading to a surface-adsorption reaching close to 100% depending on protein concentrations used^30^ and purification can further be complicated by the cross-linking and/or aggregation of cationic liposomes (DDA:TDB) with protein. We have demonstrated the capability of the filtration device to separate non-adsorbed ovalbumin (OVA) from a cationic liposome formulation and residual solvent with high liposome adjuvants recovery (87%) (Fig. 3). This presents compelling evidence that our micro continuous-flow purification device, i.e. TFF device, is capable of providing an effective post-production purification step, with the option to recycle purified protein for subsequent applications.

The liposome process flow system presented here (Fig. 4) facilitates the complete removal of the free drug, which was previously only achievable by time intensive, bench-top dialysis^18,22^. The encapsulation and solubilisation of drug with low aqueous solubility in the bilayer of liposomes has been investigated previously using a microfluidics based system^18^. In that study the assembly of PC:Chol liposomes was performed using a SHM, and the method was established as a robust, reproducible approach for preparing size-controlled liposomes as solubilising agents. The same SHM is implemented in this herein reported system to investigate the effects of continuous processing on drug encapsulation by measuring amounts of drug encapsulated in the liposome bilayer. Very importantly, the amount of encapsulated drug and physical characteristics (size, PDI) show that continuous processing and the pressures applied in the TFF have no adverse effect on liposome integrity (Table 3). The presented assembly utilizes the methanol solubilisation as the initial step of liposome production. However, it is possible to replace time-intensive production and dialysis (hours) with a micro continuous-flow system (minute-long process) manufacturing and purification to rapidly remove residual solvent. Among the main merits of using the continuous microfluidic process flow are the mild conditions during the assembly of the liposomes and the replacement of long ultracentrifugation steps for protein removal^31^. It can be concluded that the performance of the process flow system demonstrated (Fig. 4) for liposomes is consistent with: first, the results from the stand-alone SHM in terms of particle characteristic; second, the results from the stand alone TFF in terms of purification.

## Conclusions

We have successfully demonstrated for the first time the feasibility for on-chip purification of liposomal batches for process development. Liposome manufacture, drug loading and removal of contaminants (such as un-entrapped drug or protein as well as solvent residues) were performed in a continuous mode using two microfluidic devices, allowing for manufacturing, purification and concentration of liposomal drug products. These devices were successfully challenged with a range of liposomes, varying in lipid composition, surface potential, size and concentration. The results demonstrate the ability of the on-chip filtration unit to be tailored to a broad diversity of lipid-based nanoparticles by varying the operational parameters. The microfluidic devices allow for an efficient and quick investigation of several lipid or drug candidates, and meet high throughput requirements of early stage development processes. The continuous process may permit determination of liposomal characteristics (e.g. size, surface potential, particle number) and encapsulation efficiencies of a wide variety of drug molecules, allowing for future integration of process analytical technologies (PAT) to further aid reproducibility. Furthermore, the setup is of considerable interest for cost-intensive drugs or protein encapsulation development, as the process requires micro volumes. The microfluidic device developed herein can cope with a variety of proteins developed by the biopharmaceutical industry. The device has the flexibility of integrating different types of membranes to cater for a variety of uses; also has the option of scalability through parallelization of the mixer chips and TFF membranes, and thereby can be easily translated to industrial setting^32^.

## Methods

### Chemicals

Egg Phosphatidylcholine (PC), CAS: 8002–43–5, 1,2-Dipalmitoyl-*sn*-glycero-3-phospho-*rac*-(1-glycerol) sodium salt (DPPG), CAS: 67232–81–9, 1,2-Dipalmitoyl-*sn*-glycero-3-phosphocholine (DPPC), CAS: 63–89–8, and Cholesterol (Chol), CAS: 57–88–5, were obtained from Sigma-Aldrich Company Ltd. (Poole, UK). 1,2-dioleoyl-sn-glycero-3-phsphoethanolamine (DOPE), CAS: 4004–05–1, 1,2-dioleoyl-3-trimethylammonium-propane (DOTAP), CAS: 144189–73–1, dimethyldioctadecylammonium bromide (DDA), CAS: 3700–67–2, and trehalose 6,6-dibehenate (TDB), CAS: 66758–35–8 were purchased from Avanti Polar Lipids, Inc., (Alabaster, AL), purity >99% (Table I). Ethanol, CAS: 64–17–5, and methanol, CAS: 67–56–1, were obtained from Fisher Scientific (Loughborough, UK). TRIS Ultra Pure, CAS: 77–86–1, was obtained from ICN Biomedicals, Inc., (Aurora, Ohio, US). Propofol (2,6-Bis(isopropyl)phenol), CAS: 2078–54–8, and ovalbumin (chicken egg), CAS: 9006–59–1, were obtained from Sigma-Aldrich Company Ltd., (Poole, UK). Ultrafiltration regenerated cellulose membranes (p\n: U2755–10AE) were obtained from Sigma-Aldrich Company Ltd., (Poole, UK) (10 kDa, pore size 0.22 µm), and Biomax polyethersulfone ultrafiltration membrane discs with 300 kDa cutoff, pore size 0.45 µm (p\n: PBMK06210) from Merck Milipore (Darmstadt, Germany).

### Liposome batch formulations for characterisation of the Tangential-Flow Filtration (TFF) device

Multilamellar vesicles (MLV) were prepared using the lipid film hydration method^33^. Lipids were weighed and dissolved in a chloroform/methanol (9:1 v/v) mixture. Cationic liposomes comprised DDA:TDB (8:1 molar ratio) and anionic liposomes comprised DPPG, DPPC, Chol (1:1:1.3 molar ratio). The organic solvent was subsequently removed by rotary evaporation under vacuum (100 RPM, 180 mBar, Rotavapor R-100, BÜCHI Labortechnik AG, Switzerland), followed by flushing with nitrogen for removal of solvent residues (5 minutes). The thin lipid film on the bottom of a round bottom flask was hydrated with 10 mM pH 7.2 TRIS buffer. Small liposomes were formed via probe sonication (Soniprep150plus, MSE, UK; 5 min at amplitude of 5). Ethanol was manually added to the liposome formulation to a final concentration of 20% (v/v) to simulate solvent contents commonly resulting from the microfluidics production method. Ovalbumin (100 μg mL^−1^) was used as a model protein, and propofol (1 mg mL^−1^) as a model drug. These were added to the liposome formulation post-production to mimic the conditions post liposome manufacturing by microfluidics.

### Device fabrication

As previously reported, the filtration system was designed to seal membranes in place by means of mechanical clamping^24^. Two poly(methylmethacrylate) (PMMA) plates, with a straight channel (1 mm width, 1 mm depth, 45 mm length) and a 1 mm hole milled at each end were clamped together using M3 screws along the edges (Torque 10 Ncm). A 1 mm wide and 0.75 mm deep cutting was used to hold the PDMS gasket in place, which was used to secure the membrane in place (Supplementary Figure S4). Different commercially available membrane sheets were cut to the required size using a CO_2_ laser marking head (Synrad Inc., Mukilteo, WA, USA). The membranes used in this set of experiments had a cut-off of 10 kDa or 300 kDa, for drug or protein filtration, respectively. The membranes were cleaned after each experiment by back-flushing with water and stored inside the TFF system in 0.8 M saline solution, ready for the next experiment.

Additionally, a clamping system was made from PMMA (two plates held together by screws [M3]) for the staggered herringbone micromixer (SHM) chip using a micromilling machine (M3400E, Folken IND, Glendale, USA). The gasket for the filtration unit was manufactured from poly(dimethylsiloxane) (PDMS, Sylgard 184, Dow Corning, Midland, USA), according to the manufacturer’s instructions and cast in PMMA moulds, manufactured as described above. Interconnect ports (milled from 5 mm PMMA), with two holes tapped with an M3 thread were used for connection to the filtration unit; an M6 threaded hole was used for standard connection fittings (P-221, Upchurch Scientific, Oak Harbor, WA, USA).

### Backpressure regulation

Backpressures were regulated through capillaries, which were attached to the retentate outlet of the filtration device (see Supplementary Fig. S3). These capillaries restricted the flow as they were selected with internal diameters smaller than the polytetrafluoroethylene (PTFE) tubing (1/16 in. × 0.031 in., p\n: 58700-U, Sigma- Aldrich Int.) which connected the TFF device with auxiliary pumps and collection vials. Backpressure was calculated using Hagen-Poiseuille’s Law

1 $${\rm{\Delta }}P=\frac{128\cdot \mu \cdot L\cdot Q}{\pi \cdot {D}^{4}}$$

where µ, L, d and Q are the dynamic viscosity of the medium at 25 °C, the length and internal diameter of the restricting capillary, and the volumetric flow rate, respectively. We used Hagen-Poiseuille’s equation (1) to select the capillary sizes and the flow rates to attain the backpressure range from 5 to 80 psi. For each backpressure analysis, a capillary was connected to the TFF retentate outlet using PTFE tubing, ferrules (p\n: P-200, IDEX Europe GmbH, Germany) connectors (Flangeless Nuts, p\n: P-247, PEEK, M6 Flat-Bottom, for 1/16 in. OD, IDEX Europe GmbH, Germany) and metric unions (Metric Union, M6 Port, p\n: P-602, IDEX Europe GmbH, Germany). The inlet of the TFF was connected through a Luer-lock fitting and polytetrafluoroethylene PTFE tubing to a single-use plastic syringe. Water was fed in the TFF device at discrete flow rates ranging from 0.01 to 2.5 mL min^−1^ attained by a syringe pump (Nemesys, Cetoni GmbH, Germany). Backpressures were measured experimentally with a pressure sensor (40PC100, Honeywell, NJ, USA) connected on the retentate side; the data was logged with a LabVIEW virtual instrument (National Instruments, TX, USA). We compared the theoretical backpressures from equation (1) to the measured backpressures, and the measured values exceeded their calculated counterparts from 20% to 6.25% when increasing the applied backpressure from 5 to 80 psi, respectively (Supplementary Figure S5, and Supplementary Table S6). One of the TFF outlets was intentionally sealed with a flat bottom plug (p\n: P-314, M6, IDEX Europe GmbH, Germany) while a single outlet connected through a ferrule (p\n: P-200), nut (p\n: P-247) and tubing into a collection vial for liquid passing through the membrane.

### Filtration

Filtration was performed in diafiltration mode to investigate the liposome behaviour in the established pressure and flow rate range (Table 2). For this experiment, bench-top prepared liposomes in aqueous solution were spiked with drug, protein or solvent, and were introduced into the TFF by means of syringe pumps (Nemesys, Cetoni GmbH, Germany), connectors and capillaries as described earlier. A capillary was connected to the TFF, in *cis-*configuration (on the same side of the membrane), and closed the loop of the retentate fluidic line (see Supplementary Fig. S3). Retentate from the TFF was collected in an intermediate collection vial and could be injected in the device hence allowing for multiple passes, referred in this article as diafiltration cycles. Transmembrane pressure was attained by controlling the flow rates in the pump; also by adding a constriction capillary of known geometry, *i*.*e*. internal diameter and length. Retentate and permeate fractions were collected in Eppendorf tubes, assessed by weight, and used for further analysis, *i*.*e*. zeta potential, size, polydispersity, quantification via HPLC. A volume of TRIS buffer, 10 mM pH 7.2, was added after each diafiltration cycle to compensate for the amount of liquid passing through the membrane (in permeate) and to sustain steady concentration levels (in retentate) during the continuous purification process.

### Continuous process flow configuration

To test the continuous processing of liposome formation followed by liposome purification, a SHM and a TFF device were connected in sequence. The SHM (Precision Nanosystems Inc., Vancouver, Canada) consisted of two inlets, a bifurcated channel with herringbone structures, and single outlet moulded in PDMS. The channels were 200 μm in width and 79 μm in height with herringbone features of 50 μm in width, 31 μm in height, 45° angle, asymmetry index 2:1 (according to Precision Nanosystems, Inc.). Luer-lock fitting and polytetrafluoroethylene (PTFE) tubing (1/16 in. × 0.031 in., Sigma- Aldrich Int.) were used to link disposable 1 mL syringes with the two inlet ports of the chip; flow rates and flow rate ratios were controlled by syringe pumps (Nemesys, Cetoni GmbH, Germany) and the whole system was primed with Tris buffer (10 mM, pH 7.2) prior to operation. Organic phase, a weighed amount of lipids in ethanol, was injected into the first inlet of the SHM device, while in the second inlet aqueous phase (TRIS buffer, 10 mM, pH 7.2) was injected. The micromixer was held in place using a clamping device made out of PMMA. The micromixer was connected to the tangential flow filtration unit *via* an intermediate collection vial (2.0 mL Eppendorf) for additional functionality. This allows the addition of various components such as of microfluidics-manufactured liposomes prior to the filtration system for purification. A bi-directional milliGAT pump (VICI Valco, Valco Instruments Co.) was connected in-line with the retentate loop of the TFF through a capillary at the bottom of that intermediate collection vial. Transmembrane pressures was varied by restricting the flow of the retentate using different small diameter capillaries connected in-line with the TFF outlet. The retentate flowed through the capillary and was collected in the intermediate vial, while permeate passed through the membrane and was gathered in a separate tube. Both fractions were analysed for content of liposomes, propofol, protein, lipid and ethanol.

Two different liposome formulations were produced using the coupled SHM to TFF systems. For the preparation of neutral liposomes, PC and Chol (4:1 molar ratio) in ethanol were injected into the micromixer at a total flow rate (TFR) of 2 mL min^−1^ and a flow rate ratio (FRR) of 1:3; bilayer drug loading was achieved by including 1 mg mL^−1^ of propofol in the solvent stream. For the preparation of a cationic liposome formulation, DOPE and DOTAP (1:1 molar ratio) in ethanol were injected into the micromixer at a TFR of 2 mL min^−1^ at FRR 1:3. After formulation, the required amount of protein (ovalbumin, 100 μg mL^−1^) was added to the intermediate collection vial. Manually adding fresh solution to the intermediate collection vial compensated for liquid passing through the membrane into permeate. Otherwise, the amount of the fluid in the system would fall below a critical level and purification would need to be interrupted because of insufficient circulating liquid volume.

### Measurement of particle characteristics

Nanoparticle tracking analysis (NTA) was performed with a Nanosight LM20 (NanoSight, Amesbury, UK), connected to a microscope (with 20 × magnification). Liposomes were diluted 1:10 to 1:100 in distilled water, to achieve an optimal particle concentration of 10^7^–10^9^ particles/mL during measurement. NTA analysis was used to determine the particle concentration per millilitre (P/mL), recording time was 60 seconds and camera settings (shutter and gain) were adjusted manually to maximise resolution. Dynamic light scattering (DLS) (Malvern Zetasizer Nano-ZS, Malvern Instruments, Worcestershire, UK) was used to report the z-average (intensity based mean particle diameter), and to report the polydispersity (PDI), in order to assess the width of the particle distribution. Liposomes were diluted 1:10 in distilled water and measurements took place at 25 °C. Zeta potential (ZP) was measured using particle electrophoresis (Malvern NanoZS, Malvern Instruments, Worcestershire, UK).

### Propofol quantification

Quantification of propofol was performed by reverse phase HPLC (Luna 5 µ C18, Phenomenex, UK, pore size of 100 Å, particle size of 5 µm) at 268 nm. The flow rate was constant at 1 mL min^−1^ throughout with a gradient elution from 5:95 (Methanol: 0.1% Trifluoroacetic Acid, TFA, in water) to 100:0 (Methanol: 0.1% TFA in water) over 10 minutes. HPLC-grade solvents were used, sonicated and filtered. The column temperature was controlled at 35 °C. All analysis was made with Clarity, DataApex, version 4.0 (DataApex, Prague, Czech Republic). For quantification, established calibration curves of propofol were used as reported previously^14^.

### Protein and lipid quantification

Samples were loaded on a HPLC and elution was performed with a gradient from 5:95 to 100:0 (Methanol: 0.1% TFA in water) over 20 and 40 minutes for protein and lipid detection, respectively. Quantification was performed by an evaporative light scattering detector (ELSD) (Sedex 90, Sedere, France), set at 52 °C and coupled to the HPLC as described previously^18^. A calibration curve was established from standards (ovalbumin in TRIS buffer, pH 7.2, lipids in ethanol) in six replicates at concentrations from 5 to 100 μg mL^−1^ (protein) and 0.05 to 1.5 mg mL^−1^ (lipids).

### Ethanol quantification

Solvent concentration was quantified by gas chromatography (GC) using a flame ionization detector (CSi 200 Series, column TRACE 15 m × 0.25 mm × 0.25 µm TR-5, Thermo Scientific, UK), with detector temperature 230 °C, injector temperature 200 °C and an injection volume of 1 µL. The carrier gas was helium at 15 psi inlet pressure. A calibration curve (6 standards ranging from 0.5–50% v/v) was established and used for quantification using an internal reference standard (propan-1-ol). All analysis was made in Clarity DataApex version 2.4 (see above).

### Statistical analysis

Unless stated otherwise, results were reported as the mean ± one standard deviation (s.d., n = 3). One- or two-way analysis of variance (ANOVA) was used to assess statistical significance, followed by Tukey’s multiple comparing test (post-hoc analysis). A t-test was performed for paired comparisons. Significance was acknowledged for p values lower than 0.05, marked with and asterisk (*). All calculations were made in GraphPad Prism version 6.0 (GraphPad Software Inc., La Jolla, CA, US).

## Electronic supplementary material

Electronic Supplementary Information
