# Author Correction: Formation and purification of tailored liposomes for drug delivery using a module-based micro continuous-flow system

**DOI:** 10.1038/s41598-018-25217-x

**Published:** 2018-04-25

**Authors:** Nikolay Dimov, Elisabeth Kastner, Maryam Hussain, Yvonne Perrie, Nicolas Szita

**Affiliations:** 1 Department of Biochemical Engineering, University College London, London, WC1H 0AH UK; 2 Aston Pharmacy School, School of Life and Health Sciences, Aston University, Birmingham, B4 7ET UK; 3 Strathclyde Institute of Pharmacy and Biomedical Sciences, University of Strathclyde, Glasgow, G4 0RE Scotland

Correction to: *Scientific Reports* 10.1038/s41598-017-11533-1, published online 21 September 2017

This Article contains an error in legend of Figure 1.

“Particle size and polydispersity as a function of increasing backpressures in the TFF system as collected on the retentate side of the membrane. Images from NTA analysis, verifying particles in permeate (top) and retentate (bottom) stream at increasing backpressures. Particles were found in the permeate at backpressures exceeding 75 psi. All experimental datasets are presented as mean and standard deviation (mean ± s.d.) resulting from three independent runs (n = 3).”

should read:

“Particle size and polydispersity as a function of increasing backpressures in the TFF system as collected on the retentate side of the membrane. Images from NTA analysis, verifying particles in retentate (top) and permeate (bottom) stream at increasing backpressures. Particles were found in the permeate at backpressures exceeding 75 psi. Particle size and PDI was measured across a range of backpressures in both the retentate (upper) and permeate (lower) graphs. All experimental datasets are presented as mean and standard deviation (mean ± s.d.) resulting from three independent runs (n = 3).”

Additionally, this Article contains an error in the legend of Figure 2.

“(**A**) Vesicle size, polydispersity (PDI), zeta potential (ZP) and particle concentration (P/mL) for cationic (DDA:TDB) and anionic (DPPG:DPPC:Chol) liposomes before and after the TFF purification. (**B**) Images from NTA show vesicles present in the retentate side only. (**C**) Propofol and ethanol removal achieved over three diafiltration cycles for anionic liposomes (DPPG:DPPC:Chol), expressed as a percentage of the initial amount of contaminants present.”

should read:

“Vesicle size, polydispersity (PDI), zeta potential (ZP) and particle concentration (P/mL) for (**A**) cationic liposomes (DDA:TDB) and anionic liposomes (DPPG:DPPC:Chol) prior and after the TFF purification. (**B**) Non-incorporated propofol and ethanol content achieved over three diafiltration cycles for anionic liposomes (DPPG:DPPC:Chol), expressed as a percentage of the initial amount of contaminants present. (**C**) Vesicle size, polydispersity (PDI), zeta potential (ZP) and particle concentration (P/mL) for anionic liposomes (DPPG:DPPC:Chol) prior and post OVA-addition (ovalbumin, 100 μg mL^−1^), and particle characteristics after the TFF purification. (**D**) Protein (ovalbumin) and ethanol removal achieved over three diafiltration cycles for anionic liposomes, expressed as a percentage of the initial amount of contaminants present. All experimental datasets are presented as mean and standard deviation (mean ± s.d.) average of three independent runs (n = 3).”

Furthermore, this Article contains an error in the legend of Figure 3.

“Vesicle size, polydispersity (PDI), zeta potential (ZP) and particle concentration (P/mL) for (**A**) anionic liposomes (DPPG:DPPC:Chol) and (**B**) cationic liposomes (DDA:TDB) prior and post OVA-addition (ovalbumin, 100 μg mL^−1^), and particle characteristics after the TFF purification. Protein (ovalbumin) and ethanol removal achieved over three diafiltration cycles for (**C**) anionic and (**D**) cationic liposomes, expressed as a percentage of the initial amount of contaminants present. All experimental datasets are presented as mean and standard deviation (mean ± s.d.) average of three independent runs (n = 3).”

should read:

“(**A**) Vesicle size, polydispersity (PDI), zeta potential (ZP) for cationic (DDA:TDB) liposomes prior and post OVA-addition (ovalbumin, 100 μg mL^−1^), and particle concentration (P/mL) before and after TFF purification. The images from NTA show vesicles present in the retentate side only. (**B**) Ovalbumin and ethanol removal achieved over three diafiltration cycles for cationic liposomes (DDA:TDB), expressed as a percentage of the initial amount of contaminants present. All experimental datasets are presented as mean and standard deviation (mean ± s.d.) average of three independent runs (n = 3).”

Finally, this Article contains errors in the legend of Figure 5.

“Lipid recovery in the continuous liposome factory-on-a-bench for (**A**) lipid recovery after four diafiltration cycles. (**B**) Lipid concentration in four concentration cycles, related to the initial amount of lipids present prior to the concentration cycles. All experimental datasets are presented as mean and standard deviation (mean ± s.d.) average of three independent runs (n = 3).”

should read:

“Lipid recovery in the continuous liposome factory-on-a-bench for (**A**) lipid recovery after four diafiltration cycles. (**B**) Lipid concentration in four concentration cycles, related to the initial amount of lipids present prior to the concentration cycles. The data shown relates to the DOPE:DOTAP liposome formulation (1:1 molar ratio, Table 1) with ovalbumin. All experimental datasets are presented as mean and standard deviation (mean ± s.d.) average of three independent runs (n = 3).”

