# Supplementary material for: Formation and purification of tailored liposomes for drug delivery using a module-based micro continuous-flow system
Source: Sci Rep. 2017 Sep 21;7:12045. doi: 10.1038/s41598-017-11533-1 (PMC5608873; doi:10.1038/s41598-017-11533-1)
Supplement: Supplementary file 1 — Electronic Supplementary Information [file 41598_2017_11533_MOESM1_ESM.doc]

**Supplementary Information**

Formation and purification of tailored liposomes for drug delivery using a module-based micro continuous-flow system

Nikolay Dimov^1,+^, Elisabeth Kastner^2,+^, Maryam Hussain^3^, Yvonne Perrie^3^, and Nicolas Szita^1,*^

^1^Department of Biochemical Engineering, University College London, London, WC1H 0AH, UK

^2^Aston Pharmacy School, School of Life and Health Sciences, Aston University, Birmingham, B4 7ET, UK

^3^Strathclyde Institute of Pharmacy and Biomedical Sciences, University of Strathclyde, Glasgow, G4 0RE, Scotland

*n.szita@ucl.ac.uk

+these authors contributed equally to this work

| 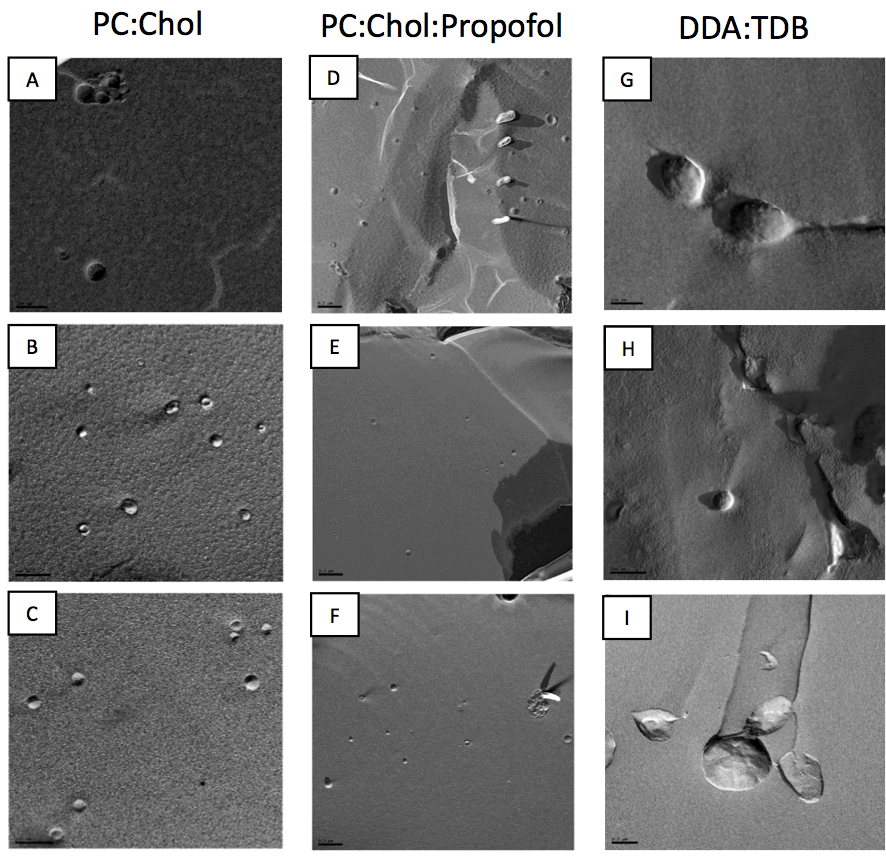 |
| --- |

| **Figure S1:** Freeze fracturing electron microscopy images of PC:Chol liposomes manufactured by microfluidics (TFR 2 mL/min, FRR 1:3) (A-C) also PC:Chol liposomes loaded with propofol manufactured by microfluidics (TFR 2 mL/min, FRR 1:3, 1 mg/mL propofol in solvent stream) (D-F) and DDA:TDB liposomes manufactured by microfluidics (G-I) (TFR 2 mL/min, FRR 1:3). The liposome suspension (2 μL) were placed in a ridged gold specimen support, frozen rapidly by plunging into a briskly stirred mixture of propane:isopentane (4:1) and cooled in a liquid nitrogen bath. Fracturing was performed with a cold knife, and replication used a Balzers BAF 400D apparatus (Forge et al., 1978, 1989). Prior to mounting the replicas on grids for electron microscopy, replicas were floated off on water, cleaned in domestic bleach, diluted 1:1 in distilled water and then washed several times in distilled water. Replicas were viewed in a transmission electron microscope (JEOL 1200EXII), which operated at 80 kV and digital images collected with a Gatan camera. Images of the freeze-fractured samples were presented in reverse contrast, where the shadows appear black. |
| --- |

**
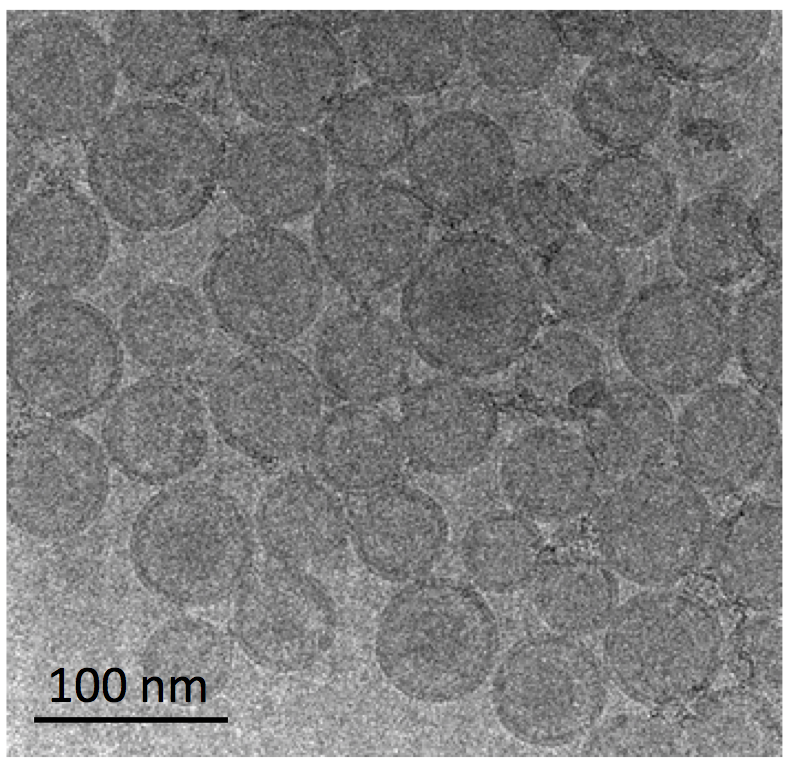
**

**Figure S2:** Cryo-TEM images of PC:Chol liposomes loaded with propofol manufactured by microfluidics (TFR 2 mL/min, FRR 1:3, 1 mg/mL propofol in solvent stream), as for Figure S1 (D-F). Images were taken on a Jeol 2011 with a 200kv beam using minimal dose protocol; scanned at low magnification and jumped to high magnification without exposing the sample to the beam first. Camera used was a Gatan ultrascan (2k by 2k pixels). Grids were lacey carbon, 200 mesh and were prepared by adding 8 microlitres of sample to a glow discharged grid, blotting from both sides for approximately 5 seconds then plunging into nitrogen cooled ethane propane mix (70% ethane). Evaluation was performed at 15,000x magnification.

**Supplementary Information S3:** Average shear rate calculation inside the retentate channel.

The average shear rate for a channel with rectangular cross-section can be calculated as


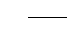


H is the height of the channel (m),
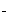
 is the pressure drop (Pa), L is the channel length (m), and
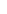
 is the viscosity of the fluid in (Pa∙s). For the retentate channel with square cross section the pressure drop is calculated according to Hagen Paiseuille’s equation


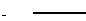


here Q is the volumetric flow rate (m^3^ s^-1^), and **a** is the side of the square (m). Hence for the highest flow rate (Q = 2.5∙10^-6^ m^3^ min^-1^, **a** = 0.001 m, L = 0.047 m) the pressure drop in Pa is:


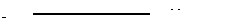


Substituting the pressure drop in the first equation gives the average shear stress inside the retentate channel at the max flow rate


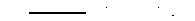


| **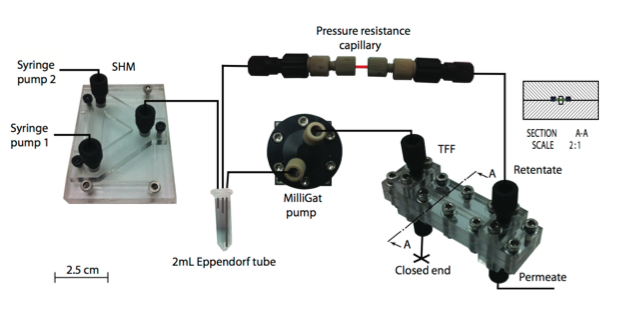** |
| --- |
| **Figure S4:** Schematic of the liposome formation and purification setup. Cross section (A-A) of the TFF with a membrane secured between two PMMA plates (hatched areas), and PDMS gasket (black) is shown. |

| 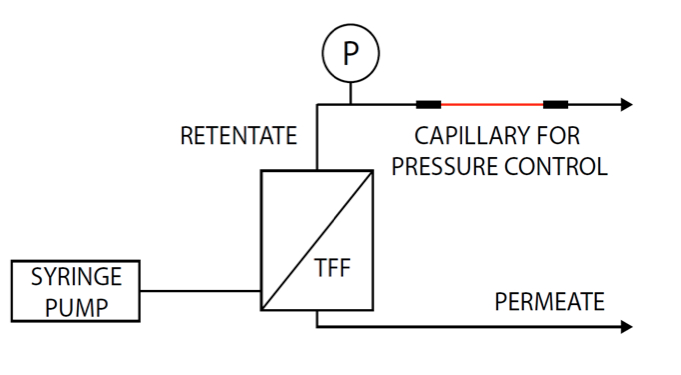 |
| --- |
| **Figure S5:** Liposome purification from batch. Liposomes prepared in batch were injected in the tangential flow filtration device using a syringe pump in a constant flow rate mode. Pressure was regulated through capillaries with different inner diameters and/or by varying the flow rates imposed by the syringe pump. Resulting pressures were continually recorded and samples were collected for analysis from the retentate and permeate outlets. |

**Table S6:** Comparison of theoretical and actual backpressures in the TFF setup at increasing flow rates from 0.01 to 0.1 mL min^-1^. Deviation in actual backpressure was extrapolated from fluctuations in the pressure recordings and expressed as ± compared to the average pressure recording (5 cm capillary I.D. 50 µm).

| **Flow rates (mL min^-1^)** | **Theoretical Backpressure (psi)** | **Actual Backpressure (psi)** |
| --- | --- | --- |
| 0.01 | 7 | 8.4 ± 1.0 |
| 0.02 | 15 | 19.5 ± 1.0 |
| 0.03 | 23 | 27.0 ± 3.0 |
| 0.05 | 39 | 55.0 ± 3.0 |
| 0.10 | 80 | 86.5 ± 5.0 |
